# The knowledge, attitudes, and perceptions towards a plant-based dietary pattern: a survey of obstetrician-gynecologists

**DOI:** 10.3389/fnut.2024.1381132

**Published:** 2024-06-04

**Authors:** Matthew J. Landry, Catherine P. Ward, Linda M. Koh, Christopher D. Gardner

**Affiliations:** ^1^Department of Population Health and Disease Prevention, Program in Public Health, University of California, Irvine, Irvine, CA, United States; ^2^Stanford Prevention Research Center, School of Medicine, Stanford University, Palo Alto, CA, United States

**Keywords:** plant-based, obstetrician-gynecologist, vegetarian, vegan, pregnant, perinatal, nutrition education

## Abstract

**Background:**

Obstetricians-gynecologists (OB/GYNs) play a critical role for their pregnant patients during their perinatal period, but research on OB/GYNs knowledge, attitudes, and perceptions regarding plant-based dietary patterns (PBDP) and how this may influence recommendations to patients is lacking. An online cross-sectional survey was conducted to examine OB/GYN’s knowledge, attitudes, and perceptions towards a PBDP.

**Methods:**

Postcards were mailed in June 2023 to a convenience sample of 5,000 OB/GYNs across the US using a mailing list provided by the American College of Obstetricians and Gynecologists. Postcards had a brief study description and a QR code that linked to an online survey asking questions about demographics, behavior (e.g., nutritional habits), and other factors that may influence knowledge, attitudes, and perceptions towards a PBDP for their patients.

**Results:**

Ninety-six OB/GYNs completed the full questionnaire (~2% response rate). Most (92%) felt that it is within an OB/GYN’s role to incorporate nutrition education and counseling within practice. However, 72% felt inadequately trained to discuss nutrition and diet-related issues with patients. Despite a perceived lack of nutrition training, 86% reported that a PBDP was safe and health-promoting, and 81% reported that a well-planned PBDP could adequately meet all nutritional needs of pregnant and lactating patients.

**Conclusion:**

Findings suggest that OB/GYNs are generally knowledgeable about the components and health benefits of a plant-based diets. However, nutrient adequacy misconceptions and lack of sufficient training to discuss nutrition with patients may result in OB/GYNs not recommending PBDPs to patients. These findings underscore the need to enhance OB/GYN graduate medical education and training by integrating education on PBDPs, therefore improving a clinician’s ability to confidently and effectively counsel pregnant persons on this aspect of perinatal care.

## 1 Introduction

General recommendations for dietary intake during pregnancy include a balanced diet meeting macro- and micronutrient needs while managing calories in line with energy expenditure and gestational weight gain recommendations (1–3). During pregnancy, a birthing person may be highly motivated to make changes to their diet to support maternal and fetal pregnancy outcomes (4, 5). However, despite the critical role of nutrition, a significant number of pregnant individuals do not meet recommended intake levels for various micronutrients, even when using dietary supplements (6). One contributing factor to this shortfall is the insufficient awareness of dietary guidelines among pregnant individuals, compounded by limited guidance from their healthcare providers (7). Without guidance, a pregnant person may seek out dietary advice from family, friends, pregnancy books, magazines, or the internet, which may not always contain reliable or accurate information (8).

Clinicians, such as obstetrician-gynecologists (OB/GYNs), have an important role in assessing the nutritional status of their pregnant patients and directing them to appropriate resources while respecting dietary choices and preferences (9, 10). Given that OB/GYNs address medical concerns throughout their patients’ lifespan and the recommendation that even healthy people visit them annually, these physicians have the opportunity to develop close relationships that other medical specialties may not. In particular, OB/GYNs play a critical role for pregnant persons who may have between 8–14 visits during the perinatal period (11), creating an opportunity for them to encourage healthy eating patterns that support a healthy pregnancy but also have the potential to influence lifelong healthy dietary habits (1).

There is strong evidence that dietary patterns high in plant foods and low in animal foods can maximize health and environmental benefits (12–15). Plant-based diets consist of a diverse family of dietary patterns, all of which encourage eating less animal foods (16). At one end of the continuum are flexitarian or semi-vegetarian diets, primarily plant-based but allowing some animal-derived food consumption, while at the other end are vegan diets, excluding all foods and beverages wholly or partly derived from animals. A commonality of all plant-based dietary patterns is inclusion of vegetables and fruits, legumes, whole grains, nuts and seeds. Several professional and medical organizations recommend dietary patterns centered around plant-based foods including the American College of Lifestyle Medicine (17), the American Heart Association (18, 19), the Academy of Nutrition and Dietetics (20), the American Cancer Society (21), and the American Institute for Cancer Research (22). A recent review of 78 clinical practice guidelines from around the world reported that 49% of guidelines advocated for dietary patterns centered around plant foods (23).

Despite the widespread acceptance and promotion of plant-based diets across a range of dietary and clinical practice guidelines (23), previous research among various subspecialities of clinicians find that some healthcare providers are hesitant to recommend a PBDP to their patients, even when appropriate.

Previous survey research within cross-sectional samples of pediatricians in both the U.S. and Israel found that a significant portion of pediatricians had a below average medical knowledge of vegetarian nutrition and did not hold positive attitudes about plant-based diets (23, 24). In a 2019 survey involving general medical providers (residents, fellows, and attendings) at one university medical center, there were mixed opinions about recommending plant-based nutrition to their current/future patients (25).

While being previously examined within several medical specialties, there remains a gap in the literature focusing specifically on knowledge, attitudes, and perceptions of plant-based dietary patterns (PBDPs) among OB/GYN practitioners. We posit that misconceptions regarding the nutritional adequacy of PBDPs, unfavorable attitudes toward them, and/or misperceptions about their suitability during the perinatal period could significantly influence the dietary advice OB/GYNs offer to pregnant patients.

## 2 Methods

Procedures for this study were followed in accordance with the ethical standards from the Helsinki Declaration and were approved by the Stanford University Human Subjects Committee (Institutional Review Board) protocol 69,628 (approved May 13, 2023). Written informed consent was obtained from the participants. This manuscript was prepared in accordance with the CROSS consensus-based reporting checklist (26).

### 2.1 Survey development and pre-testing

Survey items were taken from previous studies of medical professionals on nutrition and plant-based diets (23, 25, 27–29). Items were adapted or new items were created as needed to make them relevant to the scope and practice-based experiences of OB/GYNs. Survey items were evaluated for content validity by a group of nutritionists, registered dietitians, OB/GYNs, and academics with experience conducting surveys. Revisions were made based on comments from the review. The usability and technical functionality of the survey on the online survey platform was tested by graduate students before fielding the survey.

The final survey consisted of 43 items (multiple choice, Likert scales) focused on demographics (e.g., participant’s age, gender identity, race, ethnicity); medical education and practice characteristics (e.g., years in practice, location of practice, hours of nutrition-focused CME, role of an OB/GYN in providing nutrition care, time spent counseling on diet); personal dietary patterns (e.g., overall healthfulness, meat consumption); knowledge of components of a plant-based dietary pattern; attitudes and perceptions about the safety, nutrient adequacy, and appropriateness of plant-based dietary pattern during pregnancy and lactation; and self-efficacy to provide nutrition education and counseling focused on a plant-based dietary pattern. The researchers intentionally refrained from defining PBDPs for the participants, as a research aim was to explore respondents’ perceptions of PBDPs without introducing any preconceived notions or bias.

### 2.2 Recruitment

This study used a random, convenience sampling approach through a fee-for-use mailing list of OB/GYNs that are members of the American College of Obstetricians and Gynecologists (ACOG), the specialty’s professional membership organization for providers of women’s health care. From the ACOG mailing list of over 44,000 members, 5,000 were randomly selected and mailed a postcard which had a brief study description and a QR code. Email addresses were not available. Postcards were addressed to the OB/GYN; however, there was no indication in the mailing list if the postal address corresponded to a work or home address for the practitioner. The postcard’s QR code directed prospective survey completers to an online consent form for the survey. Because it was anticipated that some respondents would scan the QR code with their phone, the survey was designed for mobile optimization.

During informed consent, participants were told the approximate length of time of the survey, which data were stored and for how long, what mechanisms were used to protect unauthorized access, who the investigator was, and the overall purpose of the study. Following informed consent, participants advanced to the survey which was administered using Qualtrics (Qualtrics, Provo, UT).

### 2.3 Survey administration

Suggested best practices for conducting survey research among physicians were used (30). The survey was designed to take <10 min to complete, and the average response time was 9 min. The survey was designed with item-response requirements for which items were requested but not forced to be completed (i.e., the survey platform would alert a respondent about any unanswered questions but would allow a respondent to continue the survey without answering if they chose). Respondents were able to review and change their answers. To provide a logical flow through the survey, items were not randomized and adaptive questioning (certain items, or only conditionally displayed based on responses to other items) was not used. To prevent multiple participation in the study, the survey platform’s option to place a cookie on a respondent’s browser when they submitted a response was enabled. An IP address of the respondent’s computer was not captured. Participants who completed the survey had the option to be entered into a drawing to receive one of 10 gift cards valued at $50 each. The survey was available for 2 months from May 1 through June 30, 2023.

### 2.4 Statistical approach

Descriptive statistics were preformed using R Studio, version 2022.12.0 (Posit Software) to summarize survey responses.

## 3 Results

Of the 5,000 postcard invitations that were sent out to practicing OB/GYNs, approximately 7% of cards were returned as undeliverable by the postal service. A total of 110 OB/GYNs accessed the survey and completed the consent form and 96 completed at least 75% of the survey. Their responses were used in our analysis. Based on prior studies, our response rate of approximately 2% was expected for a questionnaire to medical practitioners sent via mail (25).

Respondents were distributed geographically across the U.S. and responses were collected from 30 states (including the District of Columbia) with the largest percentage of responses (17%) from California. Demographic and practice characteristics are provided in Table 1. Overall, OB/GYN respondents self-identified as primarily female, non-Hispanic white, were in practice >15 years, and practiced in a community hospital-based setting.

**Table 1:** Demographic and practice characteristics of obstetrician gynecologists to an online survey on plant-based eating patterns (*n* = 96).

Variable	*N*	%
Age		
25–34	7	7.3
35–44	33	34.4
45–54	28	29.2
55–64	20	20.8
65–74	6	6.3
75 or older	2	2.0
Gender		
Male	9	9.4
Female	84	87.5
Prefer not to say	3	3.1
Hispanic or Latino		
Yes	3	3.1
No	93	96.9
Race		
Asian	7	7.3
Black	9	9.4
White	80	83.3
Years in practice		
<5 years	15	15.6
5–15 years	32	33.3
>15 years	49	51.0
Practice environment		
Private practice	26	27.1
Academic medical center-based practice	20	20.8
Community hospital-based practice	40	41.7
Retired	1	1.0
Other	9	9.4

Of all respondents, 43% reported not following a special dietary pattern. For respondents that did follow a special dietary pattern, respondents selected multiple options that included: (15%) plant-based, (13%) pescetarian, (10%) flexitarian, (9%) vegan, (8%) vegetarian, (2%) ketogenic, (4%) paleo.

OB/GYN respondents were asked to self-rate their diet, with higher scores indicating higher quality diets and on average, self-rated diet was 7.5 ± 1.4 (range 2–10 with 10 being highest quality). Respondents’ personal consumption of meat (processed and red), poultry, dairy, and eggs are shown in Supplementary Figure S1. When asked if some people’s physiology requires them to eat meat, 40% strongly disagree, 28% somewhat disagree, 14% neither agree nor disagree, 14% somewhat agree, and 4% strongly agree.

Overall, 72% of respondents perceived that OB/GYNs were inadequately trained to discuss nutrition and diet-related issues with patients and 93% of respondents either somewhat or strongly agreed that additional training in nutrition would allow OB/GYNs to provide better clinical care to patients. When asked about the number of hours of nutrition education received in medical school, 76% reported receiving <10 h. Even following residency, few respondents reported completing nutrition-focused continuing medical education. Of all respondents, 39% reported receiving 0 h of nutrition-focused continuing medical education, with 34% reported receiving 1–10 h. Despite a perceived lack of adequacy in training, incorporation of nutrition education and counseling within OB/GYN practice was seen as being within their role and scope of practice with 92% of respondents either somewhat or strongly agreeing.

We surveyed OB/GYNs to see whether they asked patients about their dietary choices and 34% of OB/GYNs reported that they sometimes, 39% often, or 21% always routinely ask. Similarly, 62% OB/GYNs respondents either often or always routinely counsel their patients about their dietary choices. When asked if recommendations for a pregnant person’s diet are no different than if they were not pregnant, 47% strongly disagreed and 44% somewhat disagreed with the statement. Time spent during a routine appointment with a pregnant person providing nutrition education and counseling about dietary choices varied with only 18% OB/GYNs reporting spending no time at all. For those OB/GYNs who did allocate time, it was often brief, with 45% spending 1–3 min, 24% spending 4–6 min, 5% spending 7–9 min, and 8% spending ≥10 min. Respondents were also asked about their estimated time required to educate their pregnant patients about a new dietary pattern and 16% believed it would take <5 min, 36% estimated 15 min, 26% estimated 20 min, and 22% estimated at least 30 min for this educational session. In routine appointments, OB/GYNs often face time constraints, making it challenging to address all aspects of their patients’ health. To enhance patient care, OB/GYNs have the option of referring individuals to other specialists within the medical team, including registered dietitian nutritionists. However, according to survey responses, only a small number of patients (<25%) are referred to nutritionists or registered dietitian nutritionists for additional nutrition education and counseling.

Respondents were asked about whether they have seen an increase in the number of their patients who follow a plant-based dietary pattern. Of all OB/GYNs, 6% strongly disagreed, 27% somewhat disagreed, 20% neither agreed nor disagreed, 39% somewhat agreed, and 8% strongly agreed. Nearly all (98%) of OB/GYNs surveyed correctly identified a vegan and vegetarian dietary pattern based on commonly used descriptions of the diets. However, when asked about the components that make up a plant-based diet, of which there is no consensus on definition, respondents were varied (Table 2).

**Table 2:** Obstetrician gynecologists perceptions of components of a plant-based dietary pattern (*n* = 96).

	Strongly disagree	Somewhat disagree	Neither agree nor disagree	Somewhat agree	Strongly agree
Processed foods are avoided	7%	9%	13%	23%	48%
Dairy is encouraged	43%	28%	19%	8%	2%
Animal foods are limited or excluded	0%	4%	1%	20%	75%
Oil is encouraged	6%	13%	41%	24%	16%
Eggs are limited or excluded	3%	15%	16%	29%	37%
Whole foods are encouraged	3%	1%	0%	15%	81%
Fish and seafood are limited or excluded	3%	15%	9%	31%	42%

Most (60%) surveyed OB/GYNs believed that more should be done to encourage pregnant persons to adopt plant-based dietary patterns. Also, 60% felt that pregnant person who follow a plant-based dietary pattern are healthier than those who do not follow a plant-based dietary pattern. When asked to select which of 13 popular dietary patterns they would recommend as appropriate for a pregnant person, the Mediterranean diet was picked by the largest number of OB/GYNs (92%) while a very low carbohydrate dietary pattern (i.e., ketogenic) and a very low-fat dietary pattern (i.e., Ornish) were both picked by the fewest number of OB/GYNs (2%) (Table 3). A whole-food plant-based dietary pattern was picked as appropriate by 78% of providers.

**Table 3:** Percentage of obstetrician gynecologists that would recommend popular dietary patterns as appropriate for a pregnant person (*n* = 96).

Popular diet	Percentage (%)
Mediterranean	92
Whole Food Plant-Based	78
Vegetarian	75
Pescetarian	67
Flexitarian	60
Vegan	53
Dietary Approaches to Stop Hypertension (DASH)	42
Low Glycemic Index (South Beach; Zone)	32
Paleo	23
Low Fat	18
Low Carbohydrate (Atkins)	15
Very Low Fat (Ornish)	2
Very Low Carbohydrate (Ketogenic)	2

In the survey, 86% of the surveyed OB/GYNs expressed agreement or strong agreement that a plant-based dietary pattern is a safe and health-promoting diet. Further, for a patient who is already consuming a plant-based dietary pattern, 53% of OB/GYNs strongly agreed or somewhat agreed that the patient would not need to change their diet during pregnancy and lactation. However, 30% felt that a patient would need to change their diet during pregnancy and lactation. In practice, 21% of OB/GYNs reported that they tend to recommend blood tests (for nutrient inadequacy) for patients who follow a plant-based dietary pattern more than for patients who do not follow a plant-based dietary pattern while 64% reported not recommending any different plan of care for those following a plant-based dietary pattern.

Responses from the survey underscored varying viewpoints among OB/GYNs regarding the nutritional adequacy of plant-based dietary patterns. A minority of respondents, 7%, strongly disagreed with the notion that plant-based dietary patterns are nutritionally adequate. A larger portion, constituting 33%, somewhat disagreed with the statement, 14% indicated a neutral stance, neither agreeing nor disagreeing, 25% somewhat agreed and 21%, strongly agreed. However, 81% agreed or strongly agreed that if well-planned, a plant-based dietary pattern could adequately meet the nutritional needs of pregnant and lactating persons. Results from questions asking about knowledge of nutrient adequacy of selected nutrients of potential concern when on a plant-based dietary pattern are shown in Table 4. The largest misconception about nutrient adequacy was focused on complementary proteins with 48% of OB/GYNs recommending that people following a plant-based diet be concerned about complementary proteins.

**Table 4:** Obstetrician gynecologists knowledge related to plant-based dietary pattern nutrient adequacy (*n* = 96).

	Strongly disagree	Somewhat disagree	Neither agree nor disagree	Somewhat agree	Strongly agree
Most plant-based dietary patterns are higher in dietary fiber and lower in saturated fat and cholesterol than most omnivorous diets	0%	1%	5%	31%	63%
It is possible to follow a plant-based dietary pattern and obtain adequate, quality protein	2%	2%	1%	26%	69%
A person who follows a plant-based dietary pattern does not need to be concerned with eating complementary proteins (to get all essential amino acids) at the same meal	15%	33%	17%	17%	18%
It is possible to follow a plant-based dietary pattern and obtain adequate Omega-3 fatty acids	0%	13%	5%	36%	46%
It is possible to follow a plant-based dietary pattern that provides adequate amounts of vitamin B12	5%	26%	9%	27%	33%
Although the iron stores of persons who follow a plant-based dietary pattern may be reduced, the incidence of iron-deficiency anemia is not significantly different from that in omnivores	1%	19%	27%	28%	25%
Although the calcium stores of persons who follow a plant-based dietary pattern may be reduced, the incidence of osteoporosis and fracture risk are not significantly different from that in omnivores	0%	12%	30%	28%	30%

When asked if respondents felt comfortable providing nutrition education and counseling to their pregnant patients and/or their families with infants on plant-based dietary patterns in pregnancy and infancy, 4% strongly disagreed, 27% somewhat disagreed, 11% neither agreed nor disagreed, 45% somewhat agreed, and 13% strongly agreed. Despite some OB/GYNs feeling uncomfortable nutrition education and counseling about plant-based diets, >63% said they were either extremely or somewhat likely to recommend a plant-based dietary pattern to a current or future patient. Of the remaining respondents, 22% neither likely nor unlikely to recommend and 15% were either extremely or somewhat unlikely to recommend.

## 4 Discussion

The findings of this cross-sectional survey of OB/GYNs reveal several noteworthy observations regarding the knowledge, attitudes, and practices of OB/GYN practitioners about nutrition and PBDPs when counseling pregnant patients during their perinatal period. Consistent with prior studies among a variety of medical specialties, OB/GYNs had general knowledge of PBDPs and perceived the dietary pattern positively as a safe and health-promoting diet (23–25). However, our study also found a discrepancy between the belief in the positive attributes of PBDPs and actual recommendations OB/GYNs might provide to a pregnant patient.

Although a majority of OB/GYNs acknowledged that providing nutrition education and counseling fell within the scope of their practice, we found that few routinely inquired about their patients’ dietary choices. When such inquiries were made, they often tended to be brief. Our results are similar to a study that examined knowledge surrounding nutrition in pregnancy and comfort in counseling patients on nutrition and pregnancy among OB/GYN residents (31). Hachey et al. found that only two-thirds of residents sometimes counseled patients on nutrition topics and most residents reported not knowing enough to confidently counsel patients (31). Together these findings suggest that although OB/GYNs recognize the importance of nutrition, they may prioritize addressing other immediate medical concerns over nutrition counseling or may experience time constraints limiting their capacity to ask about dietary intake (32).

The majority of OB/GYNs expressed agreement or strong agreement with the notion that a PBDP is a safe and healthy. Additionally, they believed that a well-planned PBDP could adequately fulfill the nutritional requirements of pregnant and lactating persons. However, it was noted that some OB/GYNs harbored misconceptions, particularly regarding the concept of complementary proteins. It was previously believed that plant proteins with complementary amino acid profiles should be combined within each meal to ensure adequate supply of all essential amino acids; however, evidence suggests that this is not necessary within a PBDP comprised of a wide variety of foods (33). The continued misconceptions surrounding nutrient adequacy could be linked to the limited exposure of medical students and residents to comprehensive nutrition education during their medical training (34).

While efforts to enhance nutrition-related coursework and clinical experiences in medical education programs are currently underway, medical students on average receive only about 19 h of nutrition education over 4 years (35). Even when nutrition is included within curriculum, topics such as PBDP are either minimally covered or entirely absent. Nutrition education should not be viewed as an additional topic to add to already overloaded medical training curriculum. Instead, medical education and training programs should integrate concepts related to nutrition within the existing curriculum content (35). For OB/GYNs residents, the Council on Resident Education in Obstetrics and Gynecology’s (CREOG) core educational goals include the ability to counsel pregnant patients on lifestyle modifications, including nutrition, exercise, and recommended weight gain (36). Despite this goal, the depth of coverage on nutrition during pregnancy is often insufficient (31). This can leave clinicians ill-equipped or unsure in their ability to address the nutritional needs of their patients effectively. Future efforts within medical training programs must acknowledge the current deficiencies and focus on providing nutrition-related continuing medical education for OB/GYNs residents.

An emerging popular method for helping to fill important gaps in nutrition training is through culinary medicine activities and teaching kitchens (37). For example, an elective culinary medicine education training program successfully improved medical student nutrition knowledge, skills, as well as attitudes and confidence in patient nutrition counseling (38). The American College of Lifestyle Medicine (ACLM) offers an open-source culinary medicine curriculum for health professional training programs that highlights a predominantly whole food, plant-based diet (39). An evaluation of this ACLM curriculum found that the curriculum could be delivered virtually and still achieve improvements in medical student nutrition knowledge, coaching confidence, culinary skills, and desired attitudes and behaviors (40).

Findings of this study also suggest that OB/GYNs may refer few of their patients to a registered dietitian nutritionist, a finding similar among a sample of general physicians and other healthcare providers (41). This represents a missed opportunity to leverage the expertise of specialized professionals who are trained to address the nuanced nutritional needs of pregnant persons (2). Registered dietitian nutritionists often have more time to provide in-depth knowledge and assess lifestyle recommendations that are individualized and appropriate for the patient. Future research may consider the role that shared medical appointments and interprofessional collaborative practice can play in providing nutrition education and counseling during pregnancy (42–44).

Despite generally positive perceptions and attitudes towards plant-based dietary patterns, some OB/GYNs expressed reluctance to recommend such dietary patterns to their pregnant patients. In some instances, OB/GYNs said they would advise patients following a PBDP to switch to a different diet during the perinatal period. Despite a significant percentage of clinical practice guidelines from around the world that recommend dietary patterns centered around plant foods (23), many guidelines do not focus on the specific nutritional needs of pregnant persons. In the U.S., the Academy of Nutrition and Dietetics states that a well-planned plant-based diet, adjusted for the possible risks of deficiencies, is suitable during all stages of life, including pregnancy and lactation (20). Future research should study the potential disparities between individual provider practices and established evidence-based dietary guidelines.

Prior research has shown that health professionals’ personal health behaviors may influence the recommendations they give to patients (45–47). We hypothesize that reluctance of OB/GYNs to recommend a PBDP to their patient may stem from a provider’s own personal dietary habits. Although our sample of surveyed OB/GYNs often ate meal, poultry, fish, dairy, and eggs, that appeared to be lower than what one would expect from the general American population (48). About one-third of survey respondents followed either a plant-based, vegetarian, or vegan dietary pattern. Previous research from of U.S. female physicians from the Woman Physicians’ Healthy Study found that providers who identified as vegetarian were more likely to advise patients on nutrition (45). Similarly, OB/GYNs, who follow a more PBDP may be more inclined to advocate for increased consumption of vegetables, fruits, and plant-based proteins to their patients. As societal dietary trends continue to shift, it is important to continue to study how a physician’s knowledge, attitudes, beliefs, and personal dietary preferences may influence the recommendations they give to patients about specific eating patterns.

### 4.1 Strengths and limitations, and future research

While prior studies have examined the knowledge, attitudes, and perceptions towards PBDPs among medical professions, this study adds a new perspective by focusing solely on OB/GYNs. Our response rate was approximately 2%, which is similar to a 2019 study assessing the knowledge of plant-based nutrition among of medical providers which sent questionnaires via email (25). While surveys remain a widely used, cost-effective means of assessing the knowledge, attitudes, beliefs, and practices of physicians, low response rates among are a common issue (49, 50). Completion of the questionnaire was voluntary and there were no reminders or follow-up to encourage participation. Future research may consider suggested approaches known to improve survey response including emphasizing the relevance of the topic, enlisting a familiar individual to endorse the survey, expanding survey completion options to include additional modes (e.g., telephone, interactive voice response, or face-to-face), and minimizing the survey’s completion burden by employing the briefest forms feasible (49, 51). Respondents may possibly have been motivated by interest and/or knowledge in the topic, which could have potentially biased the results. Additionally, this study’s sample includes OB/GYNs who are members of the ACOG. Results may not accurately reflect perceptions and practices of OB/GYNs who do not belong to this organization. Furthermore, although responses from practitioners in all U.S. states were not collected, the study’s sample included responses from 60% of U.S. states. Surveyed OB/GYNs self-identified primarily as female and non-Hispanic white, demographics similar to that of the OB/GYN workforce according to the ACOG (52). Building upon this research, future studies should examine the knowledge, attitudes, and perceptions towards a PBDP among other medical subspecialities. Additionally, researchers should consider other recruitment tactics to improve response rates from practitioners that include a broader spectrum of racial/ethnic as well as gender perspectives.

## 5 Conclusion

The findings from this survey suggest that OB/GYNs are generally knowledgeable about the components and health benefits of a plant-based diets and view a PBDP as a safe and healthy way of eating for a pregnant person. However, misconceptions about the nutrient adequacy of plant-based diets and lack of sufficient training to discuss nutrition and diet-related issues with patients may result in OB/GYNs not recommending such PBDPs to their patients. Insights from this survey should be viewed as a call to action to develop or refine medical education and training about PBDPs for OB/GYN practitioners.

## Data availability statement

The raw data supporting the conclusions of this article will be made available by the authors, without undue reservation.

## Ethics statement

The study was approved by Stanford University Human Subjects Committee (protocol 69,628 approved May 13, 2023). The study was conducted in accordance with the local legislation and institutional requirements. The participants provided their written informed consent to participate in this study.

## Author contributions

ML: Conceptualization, Formal analysis, Funding acquisition, Investigation, Methodology, Visualization, Writing – original draft, Writing – review & editing. CW: Conceptualization, Methodology, Writing – review & editing. LK: Conceptualization, Methodology, Writing – review & editing. CG: Conceptualization, Methodology, Resources, Supervision, Writing – review & editing.
