# Supplementary material for: The knowledge, attitudes, and perceptions towards a plant-based dietary pattern: a survey of obstetrician-gynecologists
Source: Front Nutr. 2024 Jun 4;11:1381132. doi: 10.3389/fnut.2024.1381132 (PMC11183291; doi:10.3389/fnut.2024.1381132)
Supplement: Supplementary file 1 [file Image_1.pdf]

Supplementary Material

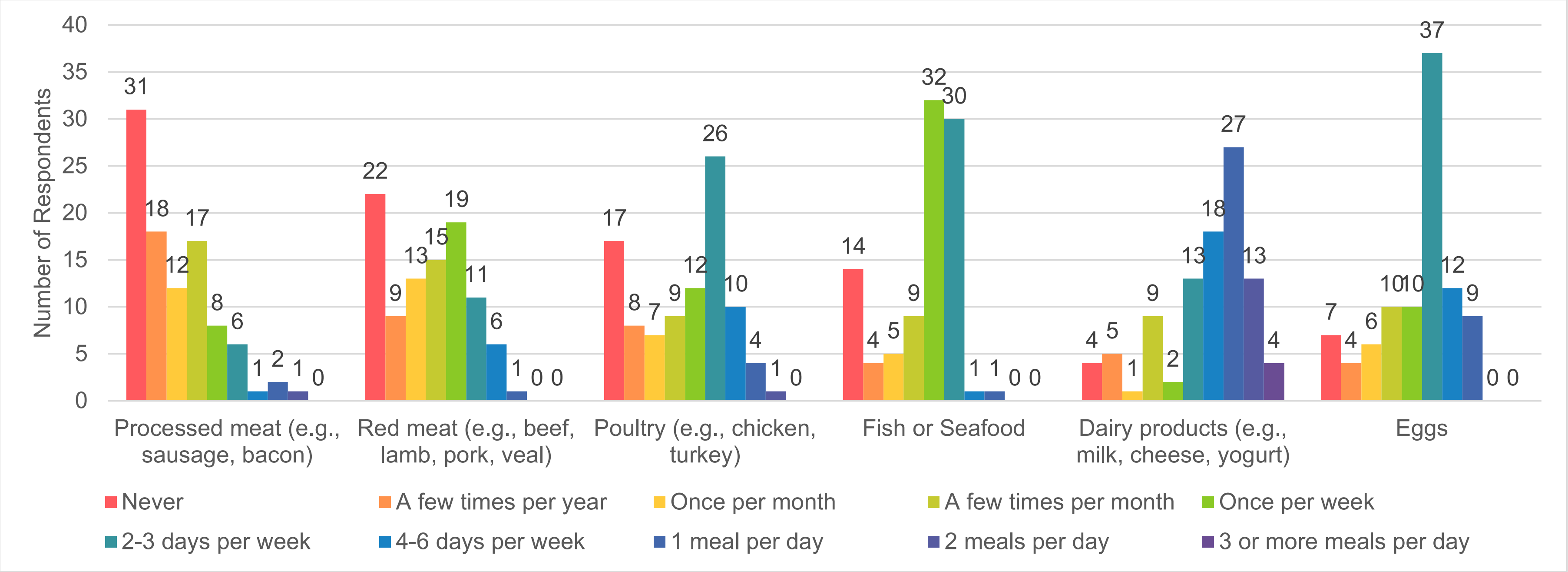

Supplementary Figure 1. Obstetrician Gynecologists Personal Meat, Poultry, Fish, Dairy, and Egg Consumption
